# Prevalence of *ABCC3*‐1767G/A polymorphism among patients with antiretroviral‐associated hepatotoxicity

**DOI:** 10.1002/mgg3.1124

**Published:** 2020-03-25

**Authors:** HariOm Singh, Sonam Lata, Ranjana Choudhari, Tapan N. Dhole

**Affiliations:** ^1^ Department of Molecular Biology National AIDS Research Institute Pune India; ^2^ Department of Clinical Epidemiology National Institute of Occupational Health Ahmedabad India; ^3^ Department of Microbiology Sanjay Gandhi Post Graduate Institute of Medical Sciences Lucknow India

**Keywords:** *ABCC3*, ARV‐associated hepatotoxicity, genetic polymorphism, HIV patients, multidrug‐resistant protein

## Abstract

**Background:**

Plasma concentrations of antiretrovirals (ARVs) regimens have considerably varied in individuals of human immunodeficiency virus (HIV) because of variations in the expression of drug‐metabolizing and transporter genes. Transporter genes play an important role in the disposition of drugs. Polymorphism in transporter gene (*ABCC3) *affects the *MRP3* expression and varies the treatment outcome.

**Method:**

We examined the polymorphism of *ABCC3*‐1767G/A gene in a total of 165 HIV patients (out of 165 HIV patients, 34 were with and 131 were without hepatotoxicity) and 156 healthy individuals using the polymerase chain reaction–restriction fragment length polymorphism method.

**Results:**

In univariate analysis, we found a decreased prevalence of* ABCC3* 1767GA, 1767GA+AA genotypes, and 1767A allele in patients with hepatotoxicity as compared to patients without hepatotoxicity (23.5% vs. 28.2% and 23.5% vs. 30.53%; 11.76% vs. 16.41%), while a higher prevalence of 1767AA genotype was observed in HIV patients in comparison with healthy controls (2.3% vs. 1.3%, odds ratio [OR] = 1.71, 95% confidence interval [CI]: 0.23–15.03, *p* = .89). The frequency of* ABCC3*‐1767AA genotype was dispersed higher in individuals with early and advanced HIV disease stage in comparison with healthy controls (5.3% vs. 1.3%, OR = 4.73, *p* = .70; 8.9% vs. 1.3%, OR = 1.89, *p* = .91). A higher occurrence of *ABCC3‐*1767AA genotype was found in tobacco using HIV patients without hepatotoxicity compared with nonusers (4.7% vs. 1.1%, OR = 4.28, *p* = .52). The distribution of *ABCC3*‐1767GA genotype was higher in nevirapine receiving HIV patients irrespective of their hepatotoxicity status as compared to nonusers (30.4% vs. 9.1%, OR = 3.34, *p* = .22; 29.4% vs. 16.7%, OR = 1.69, *p* = .77). In multivariate analysis, HIV patients receiving nevirapine and with hepatotoxicity was found to have a significant risk for severity of hepatotoxicity (OR = 4.56, 95% CI: 1.60–12.99, *p* = .004).

**Conclusion:**

*ABCC3* 1767G/A polymorphism was not significantly associated with susceptibility to ARV‐associated hepatotoxicity, although *ABCC3* 1767AA genotype designated a risk for acquisition of hepatotoxicity and advancement of the disease. Nevirapine usage emerged as an independent risk factor for hepatotoxicity severity.

## Graphical abstract

*ABCC3*‐1767G/A polymorphism is not significantly associated with susceptibility to ARV‐ associated hepatotoxicity. *ABCC3*‐1767AA genotype designated a risk for acquisition of hepatotoxicity and advancement of the disease. Nevirapine usage emerged as an independent risk factor for hepatotoxicity severity.

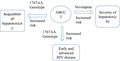

## 1 INTRODUCTION

Antiretroviral therapy (ART) is a basis for the treatment in human immunodeficiency virus (HIV) infection. However, long‐term efficacy and toxicity are the major challenges when selecting an ART regimen for the treatment of HIV. Liver is a primary organ for drug metabolism and detoxification in the body in addition to being a major target of drug toxicity (Bandara & Kennedy, 2002). High levels of antiretrovirals (ARVs) in HIV patients may cause several adverse drug reactions (ADR) like liver toxicity. Hepatotoxicity is one of the common ADR leading to the interruptions in the treatment in HIV patients (Van Dyke, Wang, & Williams, 2008). Usage of nevirapine‐based ART was associated with a higher incidence of ARV‐associated hepatotoxicity toxicity than efavirenz (Van Leth, Phanuphak, & Ruxrungtham, 2004). Reisler et al. (2001) reported that the incidence of severe hepatotoxicity was 10.8% in the efavirenz‐treated group and 8.9% in the nevirapine‐treated group. The incidence rate of nevirapine‐induced hepatotoxicity was 3.19% in India (Nagpal, Tayal, Kumar, and Gupta, 2010).

Antiretroviral therapy modifies the activity and expression of drug transporters. This could alter the drug absorption, elimination, and distribution and thereby affect the access to the target site. Multidrug resistance‐related proteins (MRP/ABCC) play an important role in elimination of numerous drugs including non‐nucleoside reverse transcriptase inhibitors (NNRTI) (Borst, Evers, & Kool, 2000). *ABCC3* plays a role in toxicological defense; it eliminates a range of (toxic) anions from hepatocytes. Studies have shown that the ARVs like atazanavir and efavirenz have significantly different plasma concentrations in HIV‐infected individuals, because of differences in the drug absorption, drug metabolism, and transport processes (Busti, Hall, & Margolis, 2004; Mukonzo, Nanzigu, & Rekić, 2011). Single nucleotide polymorphisms (SNPs) are an important factor, which influence the variation of gene expression.

Multidrug resistance‐associated protein 3 (MRP3/*ABCC3* gene) is an ATP‐binding cassette ABC transporter, expressed in adrenal gland, liver, small intestine, colon, and gall bladder in humans (Kool, Haas, and Scheffer, 1997). MRP3 is highly expressed on the basolateral membrane of enterocytes and hepatocytes, where it transports substrates into the bloodstream. *ABCC3* gene was mapped on chromosome 17q22 (Uchiumi, Hinoshita, & Haga, 1998). In the promoter region polymorphism, *ABCC3* genotype was not associated with changes in the pharmacokinetics of 4‐MUG, a substrate of MRP3 (Sasaki, Hirota, & Ryokai, 2011). The variant allele −1767A of *ABCC3* gene was significantly associated with reduction of transcriptional activity compared to the wild‐type allele, whereas a decreased expression of *ABCC3* mRNA was not detected in human liver samples (Sasaki et al., 2011). Promoter region polymorphism (−1767G/A) of *ABCC3* gene showed a significant change in *ABCC3* mRNA levels (Takechi, Hirota, & Sakai, 2018). *ABCC3*‐1767G/A polymorphism was not associated with patients with hepatocellular carcinoma (OR = 0.85, 95% CI: 0.42–1.74) (Fukuda & Kawahara, 2010). The mRNA and protein levels of MRP3 in human liver were found to vary among individuals by 86‐ and 84‐fold, respectively (Hitzl, Klein, & Zanger, 2003).

Plasma efavirenz levels vary among HIV‐infected individuals. MRP3 protein plays an important role to transport the substrates into the bloodstream. Polymorphism (−1767G/A) of *ABCC3* gene affects the expression levels, which leads to toxicological effects. Till date, the prevalence of promoter region polymorphism (−1767G/A) of *ABCC3* gene among patients with ARV‐associated hepatotoxicity has not been studied worldwide. Hence, we investigated the prevalence of *ABCC3*‐1767G/A polymorphism in two groups of HIV patients, made on the basis of the presence or absence of ARV‐associated hepatotoxicity.

## 2 MATERIAL AND METHODS

### 2.1 Subjects

The present case–control study was undertaken from October 2013 to March 2016, at the outpatient HIV clinics of the National AIDS Research Institute, Pune. The study included three groups, one consisting of 34 patients with hepatotoxicity (Grade III/IV) under NNRTI containing ART regimen, 131 HIV patients without hepatotoxicity confirmed by liver function tests (LFT), and 156 age‐matched healthy controls. Patients with hepatotoxicity due to other causes such as hepatitis B, hepatitis C, tuberculosis, and concurrent untreated opportunistic infections, immune reconstitution syndrome, and receiving any other known hepatotoxic drugs were excluded from the study. Control group comprised of age‐matched, one fifty‐six healthy individuals (two or more than two persons belonging to the same family were excluded in order to find out the true prevalence of genetic variation), who were free from HIV (serum‐negative on HIV‐ELISA test), hepatitis B, hepatitis C, and tuberculosis infections. ELISA for hepatitis C and HBsAg testing was performed using Ortho HCV ELISA test system and Murex HBsAg confirmatory (DiaSorin) ELISA, respectively. The study was approved by the institutional ethics committee, and written informed consent was obtained from all eligible participants.

Clinical research proforma/data collection was done using pretested, prevalidated questionnaire, personal interviews, and review of existing case records. Environmental exposures such as tobacco use and alcohol usage/intake for each subject were also recorded in the questionnaire.

Liver function test was done to evaluate the status of liver enzymes. Total bilirubin >3.22 mg/ml, SGOT >93.8 U/ml, SGPT >229.5 U/ml, and alkaline phosphatase >550.8 U/ml parameters defined the cases with hepatotoxicity in male gender, whereas total bilirubin >3.22 mg/ml, SGOT >163.2 U/ml, SGPT >173.4 U/ml, and alkaline phosphatase >550.8 U/ml formed the case definition for hepatotoxicity in female. Total bilirubin <1.24 mg/ml, SGOT <32 U/ml, SGPT <34 U/ml, and alkaline phosphatase <108 U/ml were the criteria for labeling/recruiting HIV‐infected but without hepatotoxicity group for both the genders. Estimation of CD4 count was done by fluorescently activated cell sorter. CD4 status was used to classify patients into different subgroups, representing the severity stages of the HIV infection. CD4 ranges from <200 cells/mm^3^ were defined as an advanced stage, 201–350 cells/mm^3^ as an intermediate stage, and >350 cells/mm^3^ onward as an early stage of HIV infection.

### 2.2 DNA extraction

A peripheral blood sample of 2 ml was obtained and stored at −70°C prior to extraction of genomic DNA, which was done from peripheral blood leukocyte pellet using the QIAamp DNA Mini Kit (QIAGEN Str. 1 40724) according to the protocol given by the manufacturer of Mini Kit.

### 2.3 Genotyping

The genotyping of *ABCC3*‐1767G/A polymorphism was done by polymerase chain reaction–restriction fragment length polymorphism in study subjects. The primer used for amplification of *ABCC3*‐1767G/A polymorphism was taken from the study carried out by Fukuda et al. (2010). PCR was performed in a total volume of 20 μl with 20 pmol of each primer, genomic DNA (100–150 ng), 10 mM deoxynucleotide triphosphates, PCR buffer containing 100 mM Tris‐HCl, pH 8.6, 50 mM KCl, 1.5 mM MgCl_2_, and 1.5 units of Taq polymerase (Bangalore Genei, India). The reaction conditions for *ABCC3*‐1767G/A were as follows: initial denaturation at 94°C for 3 min, followed by 35 cycles of denaturation at 95°C for 45 s, annealing at 57°C for 30 s, extension at 72°C for 45 s, and final extension at 72°C for 10 min. Amplified product of *ABCC3* was digested using restriction enzyme *BsmAI* (Fermentas Inc.)*. ABCC3*‐1767G/A polymorphism was genotyped in 15% polyacrylamide gel using molecular weight markers and visualized after staining with ethidium bromide. Based on sequences and location of SNP, genotypes of *ABCC3* were assigned as follows: for *ABCC3:* 281bp for −1767GG, 281 bp + 241 bp + 40 bp for 1767GA, and 241 bp + 40 bp for GG genotype. Veriti 96‐well Thermal Cycler (Applied Biosystems) was used to amplify the desired DNA. PCR products and molecular weight markers were visualized after staining with ethidium bromide. Twenty percent of samples from both patients and controls were regenotyped by another trained laboratory personnel to rule out discrepancy in genotyping reporting. Ten percent of samples were sequenced to assess the genotyping error.

### 2.4 Data analysis

The age variable was expressed as mean ± standard deviation (*SD*). The deviation from Hardy–Weinberg equilibrium in controls was analyzed using chi‐square goodness‐of‐fit test. We compared the genotype frequency between HIV patients with hepatotoxicity versus. without hepatotoxicity and HIV patients versus. healthy controls using chi‐square statistic (Fisher's exact test for theoretical cell size <5). Tobacco, alcohol usages and genotypes interactions were examined in all eligible HIV patients. Odds ratios (ORs) and 95% confidence interval (CI) were calculated by unconditional binary logistic regression. All statistical analysis was performed using SPSS software version 17.0 (SPSS), and tests of statistical significance were two‐sided and taken as significant when *P‐*value was less than 0.05.

## 3 RESULTS

The study consisted of a total of 165 HIV patients of which 34 patients had hepatotoxicity, 131 were without hepatotoxicity, and 156 were healthy controls. The mean age (years ± *SD*) of patients with hepatotoxicity, patients without hepatotoxicity, and healthy controls was 40.12 years ± 4.31, 39.42 years ± 3.42, and 37 years ± 4.35, respectively. The demographic profile of HIV patients with, without hepatotoxicity and healthy controls is shown in Table 1.

**Table 1:** Characteristics of HIV patients with, without hepatotoxicity and healthy controls

Subjects	HIV patients with hepatotoxicity (grade III and IV)	HIV patients without hepatotoxicity	Healthy controls
Number	*N* = 34	*N* = 131	*N* = 155
Mean age (range)	35.14 ± 8.96	39.29 ± 1.34	36.75 ± 8.50
Females	16 (47.05)	44 (33.58)	40 (25.80)
Males	18 (52.94)	84 (64.12)	112 (72.25)
NNRTI regimen			
Efavirenz *N* = 23	11 (32.35)	12 (9.16)	Not applicable (NA)
Nevirapine *N* = 142	23 (67.64)	119 (90.83)	Not applicable
Alcohol habit			
User *N* = 51	7 (20.58)	44 (33.58)	0
Nonuser *N* = 114	27 (79.41)	87 (66.41)	0
Tobacco habit			
User *N* = 50	23 (67.64)	27 (20.61)	0
Nonuser *N* = 115	11 (32.35)	104 (79.38)	0
CD4+ status			
<200 (*N* = 95)	16 (47.05)	41 (31.29)	NA
201–350 (*N* = 50)	17 (50)	33 (25.19)	NA
>350 (*N* = 20)	1 (2.94)	19 (14.50)	NA

Abbreviations: HIV, human immunodeficiency virus; NNRTI, non‐nucleoside reverse transcriptase inhibitors.

### 3.1 Association of genotype and phenotype

#### 3.1.1 *ABCC3*‐1767G/A polymorphism and patients with ARV‐associated hepatotoxicity

The genotype and allele frequency of *ABCC3*‐1767G/A polymorphism in between HIV patients with and without hepatotoxicity and between HIV patients with hepatotoxicity and healthy controls are shown in Table 2. The distributions of *ABCC3*‐1767GA, −1767GA+AA genotypes and −1767A allele were reduced in patients with hepatotoxicity as compared to without hepatotoxicity group (76.5% vs. 69.5%; 23.5% vs. 28.2% and 23.5% vs. 30.53%; 11.76% vs. 16.41%, respectively). Almost similar results were obtained while comparing the distribution of *ABCC3*‐1767G/A polymorphism between HIV patients with hepatotoxicity and healthy controls.

**Table 2:** Frequency distribution (%) of *ABCC3*‐1767G/A genotypes/alleles in HIV patients with and without hepatotoxicity

Genotype *ABCC3*‐1767G/A	HIV patients with hepatotoxicity *N* = 34 (%)	HIV patients without hepatotoxicity *N* = 131 (%)	*p*‐Value	OR (95% CI)
GG	26 (76.5%)	91 (69.5%)	—	1 (Reference)
GA	8 (23.5%)	37 (28.2%)	0.48	0.73 (0.30–1.77)
AA	0 (0.0%)	3 (2.3%)	NS	—
GA+AA	8 (23.5%)	40 (30.53%)	0.55	0.70 (0.27–1.80)

*ABCC3*‐1767G/A Allele	HIV patients with hepatotoxicity *N* = 68 (%)	HIV patients without hepatotoxicity *N* = 262 (%)	*p*‐Value	OR (95% CI)
G	60 (88.23%)	219 (83.58%)	—	1 (Reference)
A	8 (11.76%)	43 (16.41%)	.44	0.68 (0.28–1.60)

Genotype *ABCC3*‐1767G/A	HIV patients with hepatotoxicity *N* = 34 (%)	Healthy control *N* = 156 (%)	*p*‐Value	OR (95% CI)
GG	26 (76.5%)	104 (67.7%)	—	1 (Reference)
GA	8 (23.5%)	50 (32.1%)	.50	0.70 (0.25–1.93)
AA	0 (0.0%)	2 (1.3%)	NS	
GA+AA	8 (23.5%)	52 (33.33%)	.36	0.62 (0.24–1.55)

*ABCC3*‐1767G/A Allele	HIV patients with hepatotoxicity *N* = 68 (%)	Healthy Control *N* = 312	*p*‐Value	OR (95% CI)
G	60 (88.23%)	258 (83.22%)	—	1 (Reference)
A	8 (11.76%)	54 (17.30%)	.34	0.64 (0.26–1.48)

Note

*N*, total number of HIV patients with hepatotoxicity (34), HIV patients without hepatotoxicity (131), and healthy controls (156). Odds ratios and 95% CIs were derived from logistic regression models comparing the homozygous wild‐type genotype/allele (GG genotype and G allele for *ABCC3*‐1767G/A) with other genotypes/alleles.

Abbreviation: HIV, human immunodeficiency virus.

#### 3.1.2 *ABCC3*‐1767G/A polymorphism and HIV patients without hepatotoxicity

We have calculated the Hardy–Weinberg equilibrium in healthy control population. We found that the distribution of *ABCC3*‐1767G/A polymorphism has followed the Hardy–Weinberg equilibrium (*p* = .13) in healthy controls. The distributions of *ABCC3*‐1767G/A polymorphism in HIV patients without hepatotoxicity and healthy controls are presented in Table 3. The occurrence of *ABCC3*‐1767GG, 1767GA, 1767GA+AA genotypes and *ABCC3*‐1767A allele found almost similar among HIV patients without hepatotoxicity and healthy controls (69.5% vs. 67.7%; 28.2% vs. 32.1%; 30.53% vs. 33.33%; 16.41% vs. 17.30%, respectively). The prevalence of −1767AA genotype was higher in HIV patients without hepatotoxicity as compared to healthy controls (2.3% vs. 1.3%, OR = 1.71, 95% CI: 0.23–15.03, *p* = .89).

**Table 3:** Frequency distribution of *ABCC3*‐1767G/A genotypes/alleles in HIV patients and healthy controls

Genotype *ABCC3*‐1767G/A	HIV patients *N* = 131 (%)	Healthy Control *N* = 156(%)	*p*‐Value	OR (95% CI)
GG	91 (69.5%)	104 (67.7%)	—	1 (Reference)
GA	37 (28.2%)	50 (32.1%)	.98	0.99 (0.53–1.83)
AA	3 (2.3%)	2 (1.3%)	.89	1.71 (0.23–15.03)
GA+AA	40 (30.53%)	52 (33.33%)	.70	0.88 (0.52–1.49)

*ABCC3*‐1767G/A Allele	HIV patients *N* = 262 (%)	Healthy Control *N* = 310	*p*‐Value	OR (95% CI)
G	219 (83.58%)	258 (83.22%)	—	1 (Reference)
A	43 (16.41%)	54 (17.30%)	.86	0.94 (0.59–1.49)

Note

*N*, total number of HIV patients (34) and healthy controls (155). Odds ratios and 95% CIs were derived from logistic regression models comparing the homozygous wild‐type genotype/allele (GG genotype and G allele for *ABCC3*‐1767G/A) with other genotypes/alleles.

Abbreviation: HIV, human immunodeficiency virus.

#### 3.1.3 Association of genotype and HIV disease stages

The occurrence of *ABCC3*‐1767GA genotype was higher in individuals with early HIV disease stage compared with healthy controls (57.9% vs. 67.7%). The incidence of *ABCC3*‐1767AA genotype observed to be higher in individuals with early and advanced HIV disease stage in comparison with healthy controls (5.3% vs. 1.3%, OR = 4.73, 95% CI: 0.0–76.30, *p* = .70; 8.9% vs. 1.3%, OR = 1.89, 95% CI: 0.18–19.42, *p* = .91) (Table 4).

**Table 4:** Frequency distribution of *ABCC3*‐1767G/A genotypes in different HIV disease stages and healthy controls

Genotype *ABCC3*‐1767G/A	Healthy controls *N* = 156 (%)	Early HIV disease stage		Intermediate HIV disease stage		Advanced HIV disease stage	
		*N* = 19 (%)	OR (P)	*N* = 33 (%)	OR (P)	*N* = 79 (%)	OR (P)
GG	104 (67.7%)	11 (57.9%)	1 (Reference)	25 (75.8%)	1 (Reference)	55 (69.6%)	1 (Reference)
GA	50 (32.1%)	7 (36.8%)	1.32 (0.77)	8 (24.2%)	0.67 (0.47)	22 (27.8%)	0.83 (0.65)
AA	2 (1.3%)	1 (5.3%)	4.73 (0.70)	0 (0.0%)	NS	2 (8.9%)	1.89 (0.91)

Note

(%), frequency of subjects, odds ratios and 95% CIs were derived from logistic regression models comparing the homozygous wild‐type genotype (GG genotype and G allele for *ABCC3*‐1767G/A) with other genotypes. HIV, human immunodeficiency virus; *N*, number of subjects.

#### 3.1.4 Association of genotype–environment interaction

In patients with hepatotoxicity, *ABCC3*‐1767GA genotype was underrepresented in tobacco using as compared with tobacco nonusers (14.3% vs. 25.9%, OR = 0.55, 95% CI: 0.08–3.77, *p* = .46), while *ABCC3*‐1767AA genotype was overrepresented in tobacco using HIV patients without hepatotoxicity compared with nonusers (4.7% vs. 1.1%, OR = 4.28, 95% CI: 0.29–124.5, *p* = .52) (Table 5).

**Table 5:** Frequency distribution of *ABCC3*‐1767G/A genotypes in tobacco using HIV patients with and without hepatotoxicity

HIV patients with hepatotoxicity				
Genotype *ABCC3*‐1767G/A	Tobacco user *N* = 7 (%)	Tobacco nonuser *N* = 27 (%)	*p*‐Value	OR (95% CI)
GG	6 (85.7%)	20 (74.1%)	—	1 (Reference)
GA	1 (14.3%)	7 (25.9%)	.46	0.55 (0.08–3.77)
AA	0 (0.0)	0 (0.0%)	—	—

HIV patients without hepatotoxicity				
Genotype *ABCC3*‐1767G/A	Tobacco user *N* = 43 (%)	Tobacco nonuser *N* = 88 (%)	*p*‐Value	OR (95% CI)
GG	29 (67.4%)	62 (70.5%)	—	1 (Reference)
GA	12 (27.9%)	25 (28.4%)	.88	1.03 (0.42–2.50)
AA	2 (4.7%)	1 (1.1%)	.52	4.28 (0.29–124.5)

Note

(%), frequency of subjects, odds ratios and 95% CIs were derived from logistic regression models comparing the homozygous wild‐type genotype (GG genotype and G allele for *ABCC3*‐1767G/A) with other genotypes. HIV, human immunodeficiency virus; *N*, number of subjects.

The occurrence of *ABCC3*‐1767GA was almost similar in tobacco using HIV patients without hepatotoxicity compared with nonusers (27.9% vs. 28.4%, OR = 1.03, 95% CI: 0.42–2.50, *p* = .88). In both the groups of HIV patients (with and without hepatotoxicity), almost similar results were obtained while comparing the distribution of *ABCC3*‐1767G/A polymorphism between alcohol consuming and nonalcoholics (Table 6).

**Table 6:** Frequency distribution of *ABCC3*‐1767G/A genotypes in alcohol using HIV patients with and without hepatotoxicity

HIV patients with hepatotoxicity				
Genotype *ABCC3*‐1767G/A	Alcohol user *N* = 7 (%)	Alcohol nonuser *N* = 27 (%)	*p*‐Value	OR (95% CI)
GG	6 (85.7%)	20 (74.1%)	—	1 (Reference)
GA	1 (14.3%)	7 (25.9%)	.46	0.55 (0.08–3.77)
AA	0 (0.0)	0 (0.0%)		

HIV patients without hepatotoxicity				
Genotype *ABCC3*‐1767G/A	Alcohol user *N* = 44 (%)	Alcohol nonuser *N* = 87 (%)	*p*‐Value	OR (95% CI)
GG	29 (65.9%)	62 (71.3%)	—	1 (Reference)
GA	13 (29.5%)	24 (27.6%)	.88	1.16 (0.48–2.79)
AA	2 (4.5%)	1 (1.1%)	.52	4.28 (0.29–124.55)

Note

(%), frequency of subjects, odds ratios and 95% CIs were derived from logistic regression models comparing the homozygous wild‐type genotype (GG genotype and G allele for *ABCC3*‐1767G/A) with other genotypes. HIV, human immunodeficiency virus; *N*, number of subjects.

The distribution of *ABCC3*‐1767G/A polymorphism in HIV patients with hepatotoxicity, who are further subclassified on the basis of the receiving of two ART drugs, namely nevirapine and efavirenz, is shown in Table 7. Similar drug groupwise frequency distribution of *ABCC3*‐1767G/A polymorphism in HIV patients without hepatotoxicity is also shown in Table 7. In both the groups of HIV patients (with and without hepatotoxicity), the incidences of *ABCC3*‐1767GA genotype were observed to be increased in nevirapine users in comparison with efavirenz users (30.4% vs. 9.1%, OR = 3.34, 95% CI: 0.46–23.96, *p* = .22; 29.4% vs. 16.7%, OR = 1.69, 95% CI: 0.31–12.15, *p* = .77). While in HIV patients without hepatotoxicity, the occurrence of *ABCC3*‐1767AA genotype was decreased in nevirapine users as compared to efavirenz users (0.8% vs. 16.7%, OR = 0.05, 95% CI: 0.00–0.80, *p* = .02).

**Table 7:** Frequency distribution of *ABCC3*‐1767G/A genotypes in HIV patients receiving NNRTI regimen with and without hepatotoxicity

HIV patients with hepatotoxicity				
Genotype *ABCC3*‐1767G/A	Nevirapine users *N* = 23 (%)	Efavirenz users *N* = 11 (%)	*p*‐Value	OR (95% CI)
GG	16 (69.6%)	10 (90.9%)	—	1 (Reference)
GA	7 (30.4%)	1 (9.1%)	.22	3.34 (0.46–23.96)
AA	0 (0.0%)	0 (0.0%)	—	—

HIV patients without hepatotoxicity				
Genotype *ABCC3*‐1767G/A	Nevirapine users *N* = 119 (%)	Efavirenz users *N* = 12 (%)	*p*‐Value	OR (95% CI)
GG	83 (69.7%)	8 (66.7%)	—	1 (Reference)
GA	35 (29.4%)	2 (16.7%)	.77	1.69 (0.31–12.15)
AA	1 (0.8%)	2 (16.7%)	.02	0.05 (0.00–0.80)

Note

(%), frequency of subjects, odds ratios and 95% CIs were derived from logistic regression models comparing the homozygous wild‐type genotype (GG genotype and G allele for *ABCC3*‐1767G/A) with other genotypes. HIV, human immunodeficiency virus; *N*, number of subjects; NNRTI, non‐nucleoside reverse transcriptase inhibitors; NS, not significant.

In HIV patients without hepatotoxicity, the prevalence of *ABCC3*‐1767AA genotype was reduced in alcohol + nevirapine and alcohol + efavirenz consumers as compared to nonuser (7.89% vs. 14.82%, OR = 0.47, 95% CI: 0.09–2.16, *p* = .44; 16.67% vs. 83.33%, OR = 0.04, 95% CI: 0.0–1.29, *p* = .08) (Table 8).

**Table 8:** Frequency distribution of *ABCC3*‐1767G/A genotypes in alcohol and NNRTI regimen using HIV patients with hepatotoxicity and without hepatotoxicity

HIV patients with hepatotoxicity				
Genotype *ABCC3*‐1767G/A	Alcohol + Nevirapine user *N = *5 (%)	Alcohol nonuser + Nevirapine users *N* = 18 (%)	*p*‐Value	OR (95% CI)
GG	1 (50.0%)	4 (44.44%)	—	1 (Reference)
GA	1 (50.0%)	4 (44.44%)	.42	1.00 (—)
AA	0 (0.0%)	1 (11.12%)	NS	—

HIV patients with hepatotoxicity				
Genotype *ABCC3*‐1767G/A	Alcohol + Efavirenz user *N* = 2	Alcohol nonuser + Efavirenz user *N* = 9	*p*‐Value	OR (95% CI)
GG	0 (0.0%)	1 (11.12%)	—	1 (Reference)
GA	2 (100%)	4 (44.44%)	NC	—
AA	0 (0.0%)	4 (44.44%)	NS	—

HIV patients without hepatotoxicity				
Genotype *ABCC3*‐1767G/A	Alcohol + Nevirapine user *N* = 38 (%)	Alcohol nonUser + Nevirapine users *N* = 81 (%)	*p*‐Value	OR (95% CI)
GG	18 (47.37%)	34 (41.98%)	—	1 (Reference)
GA	17 (44.74%)	35 (43.20%)	1.00	0.92 (0.38–2.24)
AA	3 (7.89%)	12 (14.82%)	.44	0.47(0.09–2.16)

HIV patients without hepatotoxicity				
Genotype *ABCC3*‐1767G/A	Alcohol + Efavirenz users *N* = 6	Alcohol nonUser + Efavirenz users *N* = 6	*p*‐Value	OR (95% CI)
GG	0 (0.0%)	0	—	—
GA	5 (83.33%)	1 (16.67%)	—	1 (Reference)
AA	1 (16.67%)	5 (83.33%)	.08	0.04 (0.0–1.29)

Note

(%), frequency of subjects, odds ratios and 95% CIs were derived from logistic regression models comparing the homozygous wild‐type genotype (GG genotype and G allele for *ABCC3*‐1767G/A) with other genotypes. HIV, human immunodeficiency virus; *N*, number of subjects; NC, not calculable; NNRTI, non‐nucleoside reverse transcriptase inhibitors; NS, not significant.

### 3.2 Risk factors of ARV‐associated hepatotoxicity: multivariate logistic regression analysis

Relationships of age, sex, tobacco, alcohol, baseline CD4 counts, and *ABCC3*‐1767G/A polymorphism with ARV‐associated hepatotoxicity were explored by multivariate logistic regression analysis. *ABCC3*‐1767G/A polymorphism, age, sex, tobacco, alcohol usage, and baseline CD4 counts were not associated with susceptibility to ARV‐associated hepatotoxicity. While comparing between HIV patients with and without hepatotoxicity, nevirapine showed a significant risk for occurrence of severity of hepatotoxicity (OR = 4.56, 95% CI: 1.60–12.99, *p* = .004) (Table 9).

**Table 9:** Multivariate analysis between HIV patients with and without hepatotoxicity

Variables	B	S.E.	*df*	*p*‐Value	OR (95% CI)
1767GG			2	.99	
1767GA	−0.016	0.481	1	.97	0.98 (0.38–2.52)
1767AA	20.48	21728.589	1	.99	79056108 (0.0—
Age	−0.040	0.031	1	.20	0.96 (0.90–1.02)
Sex	0.374	0.464	1	.42	1.45 (0.45–4.63)
Tobacco user	0.370	0.594	1	.53	1.44 (0.370–4.003)
Alcohol user	0.377	0.611	1	.53	1.45 (0. 44–4.83)
NNRTI drug user	1.518	0.534	1	**.004**	**4.56 (1.60–12.99)**
HIV stages (intermediate)	−1.256	0.866	1	.14	0.28 (0.05–1.55)
HIV stages (advanced)	−0.720	0.843	1	.39	0.48 (0.09–2.54)

Note

*ABCC3*‐1767G/A polymorphism, age 18–50 years, sex, tobacco user, alcohol user, NNRTI drug user, baseline CD4. Significant values (<0.05) represented in bold.

Abbreviations: HIV, human immunodeficiency virus; NNRTI, non‐nucleoside reverse transcriptase inhibitors.

## 4 DISCUSSION

Antiretrovirals alter the activity and expression of active drug transporters, which in turn can affect the drug absorption, elimination, and distribution, thereby playing an important role in the treatment outcome. NNRTI (delavirdine, efavirenz, and nevirapine) interact with MRP3 in vitro. MRP3 proteins are one of the main types of transporter proteins, which play an important role in the drug transport of major drugs including efavirenz (Zhou, Di, & Chan, 2008). The genetic variation may affect the global response to treatment or causing adverse drug events. Large interindividual variabilities in the expression of transporters may lead to inappropriate drug concentrations, causing drug toxicity or insufficient therapeutic effects in the liver. Evidence has revealed that variabilities in the expression and activities of some transporters affect pharmacokinetics as well as pharmacological and/or toxicological effects (Cascorbi, 2006; Gotanda, Tokumoto, & Hirota, 2015; Ieiri, Higuchi, & Sugiyama, 2009; Maeda & Sugiyama, 2008). Genetic polymorphisms influence the gene expression. SNPs in pharmacokinetic‐related genes play an important role in interindividual variations in drug responses (Gotanda et al., 2015; Ieiri et al., 2009; Ma & Lu, 2011; Maeda & Sugiyama, 2008). Genetic variations in the *ABCC3* gene showed large interindividual variability in expression levels (Takechi et al., 2018).

In our study, the genotype/allele distribution of *ABCC3*‐1767G/A polymorphism was comparable with study carried out in the population of Caucasians and Japanese by Sasaki et al. (2011) and Fukuda et al. (2010) and dissimilar with study done in the population of African Americans and Japanese by Sasaki et al. (2011). In this study, *ABCC3*‐1767G/A polymorphism was neither associated with acquisition of ARV‐associated hepatotoxicity nor its severity. However, a higher prevalence of 1767AA genotype was found in HIV patients compared with healthy controls (2.3% vs. 1.3%, OR = 1.71). Similar results were obtained by Fukuda et al. (2010). *ABCC3*‐1767G/A polymorphism was not associated with patients with hepatocellular carcinoma (OR = 0.85, 95% CI: 0.42–1.74) (Fukuda & Kawahara, 2010).

The present study was undertaken as a case–control, and the current CD4 count was taken as a substitute for current HIV disease stage. Since the time points for HIV infection are not known, we assumed that the results may be confounded by the duration of HIV infection. In our study, *ABCC3*‐1767AA genotype was found to be higher in individuals with early and advanced HIV disease stages in comparison with healthy controls (5.3% vs. 1.3%, OR = 4.73, *p* = .70; 8.9% vs. 1.3%, OR = 1.89, *p* = .91). This suggests that the presence of −1767AA genotype may facilitate the risk of advancement of HIV disease.

The gene–environment interactions determine the pathophysiology of the disease (Deng, Newman, & Dunne, 2004). However, for case–control association studies for environmental influences, cases must have matched controls (Greenland, 1980). It is assumed that a case study is always better to look at for the effect of gene and environment. Here, we have chosen the case‐only analysis. The consumption of heavy alcohol had a negative impact on the CD4 cell counts of HIV patient's naïve to ART (Samet, Cheng, & Libman, 2007). In our study, in HIV patients without hepatotoxicity, the occurrence of *ABCC3*‐1767AA genotype was higher in alcohol users as compared to nonusers (4.7% vs. 1.1%, OR = 4.28). In HIV patients with and without hepatotoxicity, *ABCC3*‐1767GA genotype showed a risk for acquisition of hepatotoxicity and its severity in nevirapine users (29.4% vs. 16.7%, OR = 1.69; 30.4% vs. 9.1%, OR = 3.34). It supports the idea that individuals with −1767GA genotype and alcohol and nevirapine usage are more prone to develop alcohol‐ and drug‐related hepatotoxicity.

Also, in HIV patients without hepatotoxicity and either taking alcohol + nevirapine or alcohol + efavirenz, the occurrences of *ABCC3*‐1767AA genotype were reduced as compared to alcohol nonusers (7.89% vs. 14.82%; 16.67% vs. 83.33%, respectively).

While comparing HIV patients with and without hepatotoxicity, on multivariate logistic regression analysis, we found that nevirapine is as an independent risk factor for acquisition of hepatotoxicity severity (OR = 4.56, *p* = .004).

The present study has certain limitations, which are enlisted as follows: (a) It can only ascertain the association, (b) could not define the causality, (c) we had not determined the plasma efavirenz and nevirapine drug levels in our subjects, and (d) we had assigned a ratio of 1:4 for case controls. Although we could not reach up to predetermined adequate numbers in controls, our case–control ratio is about 1:3, which is also a robust ratio.

## 5 CONCLUSION

*ABCC3*‐1767G/A polymorphism was not significantly correlated with hepatotoxicity status among the HIV patients. Though −1767AA genotype showed a risk for acquisition of hepatotoxicity and advancement of HIV disease, −1767GA genotype may also increase the risk for acquisition of hepatotoxicity and its severity among the HIV patients taking nevirapine ART drug. Transporter genes play an important role in the disposition of drugs since interindividual variabilities in the expression of transporter genes may lead to inappropriate plasma drug concentrations, thereby causing drug toxicity in the liver. Polymorphisms in drug transporter genes play an important role in interindividual variations in drug responses. Hence, further study of *ABCC3*‐1767G/A polymorphism should be done in larger sample size in genetically diverse populations and the subsequent correlation of the results with plasma levels of drugs might be helpful to predict the drug‐specific response and drug efficiency.

## CONFLICT OF INTEREST

None declared.

## AUTHOR CONTRIBUTIONS

HariOm Singh: Over all supervision. Sonam Lata: Laboratory work. Ranjana Choudhari: Native English, medical and critical review of the manuscript.

## References

[mgg31124-bib-0001] Bandara, L. R. , & Kennedy, S. (2002). Toxicoproteomics– A new preclinical tool. Drug Discovery Today, 7, 411–418. 10.1016/S1359-6446(02)02211-0 11916571

[mgg31124-bib-0002] Borst, P. , Evers, R. , Kool, M. , & Wijnholds, J. (2000). A family of drug transporters: The multidrug resistance‐associated proteins. Journal of the National Cancer Institute, 92, 1295–1302. 10.1093/jnci/92.16.1295 10944550

[mgg31124-bib-0003] Busti, A. J. , Hall, R. G. , & Margolis, D. M. (2004). Atazanavir for the treatment of human immunodeficiency virus infection. Pharmacotherapy, 24, 1732–1747. 10.1592/phco.24.17.1732.52347 15585441

[mgg31124-bib-0004] Cascorbi, I. (2006). Role of pharmacogenetics of ATP‐binding cassette transporters in the pharmacokinetics of drugs. Pharmacology & Therapeutics, 112, 457–473. 10.1016/j.pharmthera.2006.04.009 16766035

[mgg31124-bib-0005] Deng, Y. , Newman, B. , Dunne, M. P. , Silburn, P. A. , & Mellick, G. D. (2004). Case‐only study of interactions between genetic polymorphisms of GSTM1, P1, T1 and Z1 and smoking in Parkinson's disease. Neuroscience Letters, 366, 326–331. 10.1016/j.neulet.2004.05.061 15288444

[mgg31124-bib-0006] Fukuda, M. , Kawahara, Y. , Hirota, T. , Akizuki, S. , Shigeto, S. , Nakajima, H. , … Ohnishi, A. (2010). Genetic polymorphisms of hepatic ABC‐transporter in patients with hepatocellular carcinoma. Journal of Cancer Therapy, 1, 114–123. 10.4236/jct.2010.13019

[mgg31124-bib-0007] Gotanda, K. , Tokumoto, T. , Hirota, T. , Fukae, M. , & Ieiri, I. (2015). Sulfasalazine disposition in a subject with 376C>T (nonsense mutation) and 421C>A variants in the ABCG2 gene. British Journal of Clinical Pharmacology, 80, 1236–1237. 25872459 10.1111/bcp.12654 PMC4631197

[mgg31124-bib-0008] Greenland, S. (1980). The effect of misclassification in the presence of covariates. American Journal of Epidemiology, 112, 564–569. 10.1093/oxfordjournals.aje.a113025 7424903

[mgg31124-bib-0009] Hitzl, M. , Klein, K. , Zanger, U. M. et al (2003). Influence of omeprazole on multi drug resistance protein3 expression in human liver. Journal of Pharmacology and Experimental Therapeutics, 304, 524–530. 10.1124/jpet.102.043547 12538803

[mgg31124-bib-0010] Ieiri, I. , Higuchi, S. , & Sugiyama, Y. (2009). Genetic polymorphisms of uptake (OATP1B1, 1B3) and efflux (MRP2, BCRP) transporters: Implications for inter‐individual differences in the pharmacokinetics and pharmacodynamics of statins and other clinically relevant drugs. Expert Opinion on Drug Metabolism & Toxicology, 5, 703–729. 10.1517/17425250902976854 19442037

[mgg31124-bib-0011] Kool, M. , de Haas, M. , Scheffer, G. L. , Scheper, R. J. , van Eijk, M. J. , Juijn, J. A. , … Borst, P. (1997). Analysis of expression of cMOAT (MRP2), MRP3, MRP4, and MRP5, homologues of the multidrug resistance‐associated protein gene (MRP1), in human cancer cell lines. Cancer Research, 57, 3537–3547. 9270026

[mgg31124-bib-0012] Ma, Q. , & Lu, A. Y. (2011). Pharmacogenetics, pharmacogenomics, and individualized medicine. Pharmacological Reviews, 63, 437–459. 10.1124/pr.110.003533 21436344

[mgg31124-bib-0013] Maeda, K. , & Sugiyama, Y. (2008). Impact of genetic polymorphisms of transporters on the pharmacokinetic, pharmacodynamic and toxicological properties of anionic drugs. Drug Metabolism and Pharmacokinetics, 23, 223–235. 10.2133/dmpk.23.223 18762709

[mgg31124-bib-0014] Mukonzo, J. K. , Nanzigu, S. , Rekić, D. , Waako, P. , Röshammar, D. , & Ashton, M. , … Aklillu, E. (2011). HIV/AIDS patients display lower relative bioavailability of efavirenz than healthy subjects. Clinical Pharmacokinetics, 50, 531–540. 10.2165/11592660-000000000-00000 21740076

[mgg31124-bib-0015] Nagpal, M. , Tayal, V. , Kumar, S. , & Gupta, U. (2010). Adverse drug reactions to antiretroviral therapy in AIDS patients at a tertiary care hospital in India: A prospective observational study. Indian Journal of Medical Sciences, 64, 245–252. 10.4103/0019-5359.99597 22885315

[mgg31124-bib-0016] Reisler, R. L. S. , Servoss, J. , & Robbins, G. , et al. (2001) The 1st IAS Conference on HIV Pathogenesis and Treatment. Buenos Aires, Argentina.

[mgg31124-bib-0017] Samet, J. H. , Cheng, D. M. , Libman, H. , Nunes, D. P. , Alperen, J. K. , & Saitz, R. (2007). Alcohol consumption and HIV disease progression. Journal of Acquired Immune Deficiency Syndromes, 46, 194–199. 10.1097/QAI.0b013e318142aabb 17667330 PMC2247363

[mgg31124-bib-0018] Sasaki, T. , Hirota, T. , Ryokai, Y. , Kobayashi, D. , Kimura, M. , Irie, S. , … Ieiri, I. (2011). Systematic screening of human ABCC3 polymorphisms and their effects on MRP3 expression and function. Drug Metabolism and Pharmacokinetics, 26, 374–386. 10.2133/dmpk.dmpk-10-rg-103 21512263

[mgg31124-bib-0019] Takechi, T. , Hirota, T. , Sakai, T. , Maeda, N. , Kobayashi, D. , & Ieiri, I. (2018). Interindividual differences in the expression of ATP‐binding cassette and solute carrier family transporters in human skin: DNA Methylation regulates transcriptional activity of the human ABCC3 gene. Drug Metabolism and Disposition, 46, 628–635. 10.1124/dmd.117.079061 29437875

[mgg31124-bib-0020] Uchiumi, T. , Hinoshita, E. , Haga, S. , Nakamura, T. , Tanaka, T. , Toh, S. , … Kuwano, M. (1998). Isolation of a novel human canalicular multispecific organic anion transporter, cMOAT2/MRP3, and its expression in cisplatin‐resistant cancer cells with decreased ATP‐dependent drug transport. Biochemical and Biophysical Research Communications, 252, 103–110. 10.1006/bbrc.1998.9546 9813153

[mgg31124-bib-0021] Van Dyke, R. B. , Wang, L. , & Williams, P. L. (2008). Toxicities associated with dual nucleoside reverse‐transcriptase inhibitor regimens in HIV‐infected children. The Journal of Infectious Diseases, 198, 1599–1608. 10.1086/593022 19000014 PMC2737265

[mgg31124-bib-0022] Van Leth, F. , Phanuphak, P. , Ruxrungtham, K. , Baraldi, E. , Miller, S. , Gazzard, B. , … Lange, J. (2004). Comparison of first‐line antiretroviral therapy with regimens including nevirapine, efavirenz, or both drugs, plus stavudine and lamivudine: A randomised open‐label trial, the 2NN Study. Lancet, 363, 1253–1263. 10.1016/S0140-6736(04)15997-7 15094269

[mgg31124-bib-0023] Zhou, S. F. , Di, Y. M. , Chan, E. , Du, Y. M. , Chow, V. D. W. , Xue, C. C. , … Duan, W. (2008). Clinical pharmacogenetics and potential application in personalized medicine. Current Drug Metabolism, 9, 738–784. 18855611 10.2174/138920008786049302

